# Explainable AI models for predicting venous thromboembolism following revascularization therapy in ischemic stroke patients: A retrospective cohort study

**DOI:** 10.1097/MD.0000000000048657

**Published:** 2026-05-15

**Authors:** Yanfeng Li, Rong Li, Qingshi Zhao, Min Gui, Yun Han, Rongjia Pan, Youli Jiang, Guisu Li

**Affiliations:** a Department of Neurology, People’s Hospital of Longhua, Shenzhen, China; b Department of Traumatic Orthopedics, Shenzhen Second People’s Hospital, Shenzhen, Guangdong Province, China; c Department of Rehabilitation Medicine, Nanjing Drum Tower Hospital, Affiliated Hospital of Medical School, Nanjing University, Nanjing, Jiangsu Province, China.

**Keywords:** acute ischemic stroke, deep vein thrombosis, machine learning, predictive modeling, SHapley Additive exPlanations

## Abstract

Deep vein thrombosis (DVT) is a serious complication in acute ischemic stroke (AIS) patients undergoing revascularization therapy, but prediction tools remain limited. To develop and validate machine learning models for predicting DVT risk in AIS patients after revascularization therapy, and to enhance clinical decision-making through model interpretability. A retrospective cohort study was conducted using data from the Shenzhen Stroke Database, including AIS patients who underwent endovascular thrombectomy and/or thrombolytic therapy. Various machine learning models, including random forest (RF), support vector machine, gradient boosting machine, decision tree, and Gaussian naive Bayes, were trained and validated using a 70:30 train-validation split. The synthetic minority over-sampling technique was applied to address class imbalance. Among 362 AIS patients undergoing revascularization therapy, DVT incidence was 8.84%. The RF model achieved the highest prediction accuracy with an area under the curve of 0.87. Key predictors included D-dimer levels, aspirin use, National Institutes of Health Stroke Scale score during hospitalization, international normalized ratio, and anti-infective treatments. SHapley Additive exPlanations analysis enhanced model interpretability, providing clear insights into individual predictor contributions. The RF model significantly improved DVT risk prediction in AIS patients post-revascularization, offering a more accurate and interpretable tool for clinical practice.

## 1. Introduction

Acute ischemic stroke (AIS) is a major public health concern, with global deaths rising from 2.04 million in 1990 to 3.29 million in 2019, and projected to reach 4.90 million by 2030.^[1]^ Endovascular thrombectomy (EVT) and intravenous thrombolysis are the primary treatments for AIS, significantly improving patient prognosis by restoring cerebral blood flow and reducing infarct size. However, these patients remain at risk for complications such as deep vein thrombosis (DVT) and muscle loss post-revascularization therapy. Among AIS patients receiving EVT and thrombolytic therapy, DVT prevalence ranges from 15.8% to 22.7%,^[2,3]^ with a 30-day mortality rate as high as 15.6%.^[4]^ Due to the insidious nature of venous thromboembolism (VTE) in stroke patients, early detection is both challenging and crucial for reducing unexpected mortality.

Gold-standard diagnostic methods for DVT, including color Doppler ultrasound and venography, are often impractical for routine screening because of equipment and environmental constraints. Instead, risk assessment scales can identify high-risk patients, allowing for timely preventive interventions that significantly reduce DVT and pulmonary embolism morbidity and mortality.^[5]^ Since the 1960s, various DVT risk assessment scales have been developed. Commonly used scales in stroke patients include the Padua, Caprini, Autar, and Wells scales.^[6–9]^ However, these scales are not specifically tailored for AIS patients undergoing revascularization therapy and have notable limitations. For instance, the Padua model, not designed for stroke, lacks comprehensive risk stratification.^[6]^ The Caprini scale, although effective for elderly stroke patients, includes factors less relevant to Chinese patients and is relatively complex.^[7]^ The Wells DVT scale, designed for outpatients, has lower predictive accuracy for hospitalized stroke patients compared with the Padua scale and requires further validation.^[9]^

Most existing DVT prediction models for AIS patients rely on traditional statistical methods, such as logistic regression, incorporating clinical and demographic predictors to estimate risk. These models report area under the curve (AUC) values ranging from 0.70 to 0.912, indicating moderate to good predictive performance.^[10]^ Nevertheless, the application of machine learning (ML) techniques for DVT prediction in AIS patients remains underexplored. While some studies have employed ML for broader stroke populations, specific models for AIS patients post-revascularization are scarce. Notable exceptions are studies that use gradient boosting classifiers and k-nearest neighbor classifiers, which enhance prediction accuracy but do not focus exclusively on the revascularization subset.^[11]^ No comprehensive ML-based DVT prediction model has been specifically validated for AIS patients undergoing revascularization therapy, a subgroup with unique risk factors resulting from physiological and procedural impacts such as vascular damage, reperfusion injury, and acute inflammatory responses.

Consequently, models for the general stroke populationmay lack the specificity required for AIS patients post-revascularization, potentially leading to inadequate risk stratification. Developing tailored ML models for this patient group is crucial, leveraging high-dimensional clinical data to capture the complex interplay of risk factors unique to post-revascularization AIS patients, enhancing predictive accuracy and enabling timely, targeted interventions. This study aims to fill this gap by developing and validating ML models specifically for DVT risk prediction in AIS patients who have undergone revascularization therapy, highlighting the potential of advanced ML techniques to improve patient outcomes and clinical decision-making in stroke care.

## 2. Methods

### 2.1. Study design and population

This study is a retrospective analysis based on a prospective stroke cohort. Data were collected from the Shenzhen Stroke Database, a nonpublic data collection platform supported and managed by the Shenzhen Municipal Health Commission. The database compiles comprehensive information on stroke patients from multiple tertiary hospitals in Shenzhen, including detailed records from the pre-hospital phase, through hospitalization, and into long-term follow-up for patients diagnosed and treated within 7 days of ischemic stroke onset. For this study, we focused on patients with AIS who underwent revascularization therapy. The inclusion criteria were patients aged ≥18 years, diagnosed with AIS through imaging (computed tomography [CT]/magnetic resonance imaging [MRI]), and having received EVT and/or thrombolytic therapy. Exclusion criteria included patients with a final discharge diagnosis other than ischemic stroke, those with missing data, hospital stays <3 days, no recanalization after EVT, prior history of VTE, and lower extremity agenesis. The primary outcome of this study was the incidence of DVT during the hospital stay in AIS patients following revascularization therapy. We employed a consecutive non-probabilitysampling method, including all eligible patients who met the inclusion criteria during the study period from October 2021 to December 2023. This approach was chosen to capture a comprehensive representation of the target population while minimizing selection bias. The study protocol was approved by the Medical Ethics Committee of Longhua People’s Hospital of Shenzhen (approval number: 2024073).

## 2.2. EVT and thrombolysis

In our research, most ischemic stroke EVT procedures are conducted under conscious sedation. For patients unable to cooperate, those with posterior circulation strokes, or those with a Glasgow Coma Scale (GCS) score below 8, rapid sequence induction and intubation are performed before EVT.^[12]^ Thrombectomy technique choice is based on vascular anatomy and operator preference, with successful reperfusion defined as a Thrombolysis in Cerebral Infarction score of 2b or 3. Thrombolysis, using recombinant tissue plasminogen activator or urokinase, is administered within guideline-recommended time windows. EVT is considered for large vessel occlusions within 6 hours of symptom onset, and up to 24 hours for selected patients based on advanced imaging criteria. Adhering to guidelines for patient selection, treatment timing, and procedural techniques is critical for optimizing outcomes, with multidisciplinary team involvement ensuring comprehensive care.

## 2.3. Diagnosis methods

In this study, we used CT and MRI techniques to diagnose acute ischemic stroke. Noncontrast CT was performed immediately upon arrival to quickly rule out hemorrhage and identify early signs of ischemia. This method is widely available and suitable for initial evaluation, but its sensitivity is not as good as MRI for early ischemia and small infarcts. MRI techniques include diffusion-weighted imaging and perfusion-weighted imaging, which are more sensitive than CT for early ischemia and help identify areas of restricted diffusion and perfusion defects, respectively. In addition, magnetic resonance angiography provides noninvasive imaging of cerebral vasculature using time-of-flight or contrast-enhanced techniques, providing detailed images without ionizing radiation. All DVT in this study was diagnosed using compression ultrasonography as the primary imaging test. This method involves applying gentle pressure to the veins of the lower extremities using a high-frequency linear transducer. The inability to compress the veins indicates the presence of thrombosis. The combination of these diagnostic methods ensures accurate and effective diagnosis of acute ischemic stroke and DVT.

## 2.4. Variables

This study collected a comprehensive set of potential predictors for DVT occurrence in AIS patients following revascularization therapy. Data collection focused on the first 3 days of hospitalization, ensuring all information was captured prior to DVT development. The variables included demographics (gender, age, ethnicity), clinical presentation features (wake-up stroke, weakness, dysarthria, conscious disturbance, headache, dizziness, convulsions), medical history (cerebral infarction, coronary heart disease, atrial fibrillation, diabetes, hypertension, smoking, alcohol consumption), clinical assessments (premorbid and post-admission modified Rankin scale [mRS], National Institutes of Health Stroke Scale [NIHSS], GCS), treatment modalities (EVT, thrombolytic therapy, medication regimens), in-hospital complications, and comprehensive laboratory parameters, including inflammatory markers, coagulation parameters, metabolic indicators, and physical measurements. This extensive variable selection enabled a thorough investigation of the multifactorial nature of DVT development in our target population.

## 2.5. Data collection and pre-processing

For sample size determination, we used the formula for estimating a population proportion:


$$\begin{array}{l} N=\frac{Z^{2}*P*(1-P)}{E^{2}} \end{array}$$

where *Z* = 1.96 (95% confidence level), *P* = .05 (5% expected DVT incidence based on literature), and *E* = 0.03 (margin of error).^[12,13]^ This calculation yielded a minimum required sample size of 203 patients. Our final sample of 362 patients substantially exceeded this requirement, ensuring adequate statistical power. The actual observed DVT incidence in our cohort was 8.84% (32/362), higher than previously reported rates, which highlights the importance of our study in this specific population of AIS patients undergoing revascularization therapy. Data export was performed by 2 data management and quality control personnel (LZ and GL) with all cases anonymized to ensure patient privacy.

Data preprocessing is a critical step in ML model development that significantly impacts model accuracy and performance. Our preprocessing pipeline addressed several key challenges in clinical data. Variables with missing outcome data were excluded from the study, and features with more than 20% missing values were also excluded to maintain data integrity. For variables with fewer missing values, the K-nearest neighbors algorithm was used for imputation, which preserves the statistical relationships between variables better than simple mean or median imputation. Continuous variables were standardized using *z*-score normalization (mean = 0, standard deviation = 1) to prevent features with larger scales from dominating the learning process – particularly crucial for distance-based algorithms such as support vector machine (SVM). We conducted systematic anomaly detection using the Tukey method, identifying outliers as values falling outside 1.5 times the interquartile range below the first quartile or above the third quartile. Identified outliers were evaluated for clinical plausibility; physiologically implausible values were removed, while clinically valid extreme values were retained. Categorical variables were transformed using one-hot encoding prior to model training, creating binary features for each category level. The dataset was split into training (70%) and testing (30%) sets, with the test set completely isolated until final evaluation to prevent data leakage, ensuring unbiased assessment of model generalizability to new, unseen data.

## 2.6. Model training and validation

We employed 5 distinct ML algorithms to predict DVT risk in AIS patients following revascularization therapy. SVM was selected for its ability to handle high-dimensional data with nonlinear relationships through kernel transformations (we tested linear, polynomial, and radial basis function kernels), particularly effective when the decision boundary between classes is complex. Gradient boosting machine (GBM), an ensemble technique that builds sequential decision trees (DTs) to correct errors made by previous trees, was chosen for its effectiveness in handling imbalanced datasets and capturing complex interactions between variables. Random forest (RF), an ensemble method that constructs multiple DTs and outputs the class that is the mode of the classes from individual trees, was included for its robustness against overfitting and ability to provide feature importance measures. DT, a nonparametric supervised learning method that creates a model predicting target values by learning simple decision rules, was selected for its high interpretability, allowing clinicians to understand the decision pathway. Gaussian naive Bayes, a probabilistic classifier based on applying Bayes theorem with strong independence assumptions between features, was included for its computational efficiency with smaller datasets and its ability to perform well even with limited training data.

To address the class imbalance problem (8.84% DVT incidence), we implemented the synthetic minority over-sampling technique (SMOTE) exclusively within the cross-validation framework. During each fold of our 10-fold cross-validation process, SMOTE was applied only to the training portion of that fold, generating synthetic DVT cases to balance the classes while keeping validation subsets untouched. This methodologically rigorous approach prevents data leakage that would occur if SMOTE were applied before splitting the data into folds. For hyperparameter optimization, we conducted a grid search with 10-fold cross-validation. Key parameters tuned included n_estimators (50–500), max_depth (3–15), min_samples_split (2–10), and min_samples_leaf (1–5) for RF; learning rate (0.01–0.2), n_estimators (50–300), max_depth (3–10), and subsample ratio (0.7–1.0) for GBM; and regularization parameter C (0.1–10), kernel type, and gamma values (0.001–1) for SVM. The hyperparameter combinations yielding the highest average AUC across validation folds were selected for the final models. Our final model evaluation was conducted on a completely separate test set that had never been exposed to SMOTE, providing an unbiased assessment of model performance on real-world data distributions.

## 3. Statistical analysis

All statistical analyses were performed using Python (version 3.8.5, Python Software Foundation) and R software (version 4.1.2, R Foundation for Statistical Computing, Vienna, Austria; license: GNU GPL v2). Categorical variables were summarized as frequencies and percentages, while continuous variables were presented as mean ± standard deviation for normally distributed data or median with interquartile range for non-normally distributed data. Normality was assessed using the Shapiro–Wilk test. For univariate analysis, differences between DVT and non-DVT groups were compared using the chi-square test or Fisher exact test for categorical variables, and the independent *t*-test or Mann–Whitney *U* test for continuous variables, as appropriate. Variables with *P* < .05 in univariate analysis were included in the multivariate logistic regression analysis to identify independent risk factors for DVT development. The Hosmer–Lemeshow test was used to assess the goodness-of-fit of the logistic regression model. Statistical significance was set at *P* < .05 (two-sided) for all analyses.

## 3.1. Model performance

Model performance was evaluated using sensitivity, specificity, accuracy, and area under the AUC. The AUC score was calculated to evaluate the overall performance of each model and used to generate the receiver operating characteristic (ROC) curve to illustrate the ability of each model to distinguish between positive and negative DVT cases. The SHapley Additive exPlanations (SHAP) value was used to explain the feature importance of the best-performing model and show the overall feature impact.

## 4. Results

### 4.1. Baseline characteristics

The study included 362 patients with AIS undergoing revascularization therapy, with a DVT incidence of 8.84%. The cohort had a mean age of 58.41 ± 14.32 years and comprised 73.74% males (*P* = .676). Most patients were of Han ethnicity (96.13%, *P* = .027). Key clinical features included limb weakness (78.18%, *P* < .001), dysarthria (67.96%, *P* < .001), and a history of cerebral infarction (16.02%, *P* = .618). The training set and validation set showed no significant differences in baseline characteristics (DVT incidence: *P* = .843; gender: *P* = .244; age: *P* = .380), ensuring comparability between the 2 groups. Key variables such as age, gender distribution, and initial stroke severity (NIHSS after onset: *P* = .904; NIHSS during hospitalization: *P* = .863) were well balanced across both sets, supporting the robustness of subsequent model development and validation processes (Tables 1 and 2).

**Table 1:** Baseline characteristics of AIS patients with and without DVT.

Variable	Category	AIS patients		*P* value
		DVT, N = 32 (%)	Without DVT, N = 330 (%)	
EVT	No	12 (37.50%)	206 (62.42%)	.008
	Yes	20 (62.50%)	124 (37.58%)	
Thrombolytic therapy	No	19 (59.38%)	237 (71.82%)	.156
	Yes	13 (40.62%)	93 (28.18%)	
Gender	Male	25 (78.12%)	242 (73.33%)	.676
	Female	7 (21.88%)	88 (26.67%)	
Age	/	61.31 ± 16.30	57.90 ± 14.12	.073
Ethnicity	Han	28 (87.50%)	320 (96.97%)	.027
	Others	4 (12.50%)	10 (3.03%)	
Wake-up stroke	No	30 (93.75%)	289 (87.58%)	.401
	Yes	2 (6.25%)	41 (12.42%)	
Limb weakness	No	18 (56.25%)	61 (18.48%)	<.001
	Yes	14 (43.75%)	269 (81.52%)	
Dysarthria	No	27 (84.38%)	119 (36.06%)	<.001
	Yes	5 (15.62%)	211 (63.94%)	
Lmpaired consciousness	No	30 (93.75%)	303 (91.82%)	.999
	Yes	2 (6.25%)	27 (8.18%)	
Headache	No	29 (90.62%)	328 (99.39%)	.006
	Yes	3 (9.38%)	2 (0.61%)	
Dizziness	No	30 (93.75%)	294 (89.09%)	.555
	Yes	2 (6.25%)	36 (10.91%)	
Convulsion	No	31 (96.88%)	329 (99.70%)	.169
	Yes	1 (3.12%)	1 (0.30%)	
Premorbid mRS	0–1	8 (25.00%)	314 (95.15%)	<.001
	2–3	7 (21.88%)	12 (3.64%)	
	4–5	17 (53.12%)	4 (1.21%)	
Abnormal electrocardiogram	No	19 (59.38%)	282 (85.45%)	.001
	Yes	13 (40.62%)	48 (14.55%)	
History of cerebral infarction	No	26 (81.25%)	278 (84.24%)	.618
	Yes	6 (18.75%)	52 (15.76%)	
Coronary heart disease	No	30 (93.75%)	308 (93.33%)	.998
	Yes	2 (6.25%)	22 (6.67%)	
Atrial fibrillation	No	28 (87.50%)	301 (91.21%)	.515
	Yes	4 (12.50%)	29 (8.79%)	
DM	No	29 (90.62%)	267 (80.91%)	.232
	Yes	3 (9.38%)	63 (19.09%)	
Hypertension	No	18 (56.25%)	143 (43.33%)	.193
	Yes	14 (43.75%)	187 (56.67%)	
Smoking	No	24 (75.00%)	224 (67.88%)	.550
	Yes	8 (25.00%)	106 (32.12%)	
Alcohol use	No	30 (93.75%)	218 (66.06%)	.001
	Yes	2 (6.25%)	112 (33.94%)	
mRS at admission	0–1	1 (3.13%)	22 (6.67%)	.110
	2–3	5 (15.62%)	95 (28.79%)	
	4–5	26 (81.25%)	213 (64.55%)	
Aspirin	No	22 (68.75%)	75 (22.73%)	<.001
	Yes	10 (31.25%)	255 (77.27%)	
Clopidogrel	No	17 (53.12%)	61 (18.48%)	<.001
	Yes	15 (46.88%)	269 (81.52%)	
Warfarin	No	29 (90.62%)	318 (96.36%)	.137
	Yes	3 (9.38%)	12 (3.64%)	
Rivaroxaban	No	13 (40.62%)	42 (12.73%)	<.001
	Yes	19 (59.38%)	288 (87.27%)	
Sulfonylureas	No	31 (96.88%)	296 (89.70%)	.342
	Yes	1 (3.12%)	34 (10.30%)	
Calcium antagonist	No	11 (34.38%)	113 (34.24%)	.998
	Yes	21 (65.62%)	217 (65.76%)	
Anti-infective	No	17 (53.12%)	285 (86.36%)	<.001
	Yes	15 (46.88%)	45 (13.64%)	
Traditional medicine	No	20 (62.50%)	325 (98.48%)	<.001
	Yes	12 (37.50%)	5 (1.52%)	
Complications	No	16 (50.00%)	87 (26.36%)	.007
	Yes	16 (50.00%)	243 (73.64%)	
Lipid medicine	No	25 (78.12%)	314 (95.15%)	.002
	Yes	7 (21.88%)	16 (4.85%)	
Antiplatelet therapy	No	15 (46.88%)	35 (10.61%)	<.001
	Yes	17 (53.12%)	295 (89.39%)	
Anticoagul therapy	No	25 (78.12%)	60 (18.18%)	<.001
	Yes	7 (21.88%)	270 (81.82%)	
Lipid-lowering therapy	No	7 (21.88%)	14 (4.24%)	.001
	Yes	25 (78.12%)	316 (95.76%)	
Antihypertensive therapy	No	19 (59.38%)	177 (53.64%)	.581
	Yes	13 (40.62%)	153 (46.36%)	
Antidiabetic therapy	No	30 (93.75%)	248 (75.15%)	.015
	Yes	2 (6.25%)	82 (24.85%)	
Intracranial artery stenosis ≥70%	No	28 (87.50%)	223 (67.58%)	.025
	Yes	4 (12.50%)	107 (32.42%)	
Stroke-related pneumonia	No	28 (87.50%)	276 (83.64%)	.801
	Yes	4 (12.50%)	54 (16.36%)	
Post-stroke epilepsy	No	30 (93.75%)	307 (93.03%)	.997
	Yes	2 (6.25%)	23 (6.97%)	
TOAST	Large vessel occlusion	1 (3.12%)	61 (18.48%)	<.001
	Small vessel occlusive stroke	9 (28.12%)	141 (42.73%)	
	Cardioembolic stroke	2 (6.25%)	64 (19.39%)	
	Other causes of stroke	12 (37.50%)	62 (18.79%)	
	Unexplained stroke	8 (25.00%)	2 (0.61%)	
NIHSS at onset	0–4	3 (9.38%)	87 (26.36%)	.171
	5–14	23 (71.88%)	184 (55.76%)	
	15–20	3 (9.38%)	36 (10.91%)	
	21–42	3 (9.38%)	23 (6.97%)	
NIHSS during hospitalization	0–4	4 (12.50%)	91 (27.58%)	.312
	5–14	20 (62.50%)	167 (50.61%)	
	15–20	5 (15.62%)	49 (14.85%)	
	21–42	3 (9.38%)	23 (6.97%)	
GCS	13–15	7 (21.88%)	31 (9.39%)	.027
	9–12	1 (3.12%)	49 (14.85%)	
	3–8	24 (75.00%)	250 (75.76%)	
SBP	/	142.94 ± 22.57	153.04 ± 26.43	.039
DBP	/	85.72 ± 16.15	91.47 ± 16.18	.044
Height	/	166.71 ± 6.80	165.81 ± 8.11	.709
Weight	/	69.09 ± 11.07	65.57 ± 12.54	.045
White blood cell count	/	8.90 ± 3.91	8.89 ± 3.68	.936
Neutrophil count	/	6.33 ± 3.17	6.26 ± 4.18	.537
Lymphocyte count	/	1.87 ± 1.34	2.04 ± 1.06	.105
hsCRP	/	24.34 ± 25.81	8.10 ± 15.50	<.001
INR	/	1.25 ± 0.41	1.07 ± 0.67	<.001
Fibrinogen	/	3.07 ± 0.71	3.10 ± 0.82	.543
D-dimer	/	968.61 ± 4006.78	71.36 ± 809.30	.001
Fasting blood glucose	/	6.49 ± 1.81	6.42 ± 2.49	.198
Glycosylated hemoglobin	/	5.98 ± 0.79	6.42 ± 1.69	.237
Alanine aminotransferase	/	25.01 ± 13.67	25.01 ± 21.75	.383
LDL-C	/	2.64 ± 0.78	2.95 ± 0.95	.052
Serum uric acid	/	403.51 ± 183.90	394.47 ± 113.30	.701
Serum creatinine	/	85.17 ± 30.64	83.12 ± 51.92	.293
Urea nitrogen	/	6.09 ± 2.25	7.32 ± 18.80	.281
Homocysteine	/	17.75 ± 9.00	15.49 ± 8.79	.167

Categorical variables are presented as n (%) and compared using chi-square or Fisher exact tests. Continuous variables are presented as mean ± standard deviation and compared using *t* tests or Mann–Whitney *U* tests. *P* < .05 was considered statistically significant.

AIS = acute ischemic stroke, DM = diabetes mellitus, DBP = diastolic blood pressure, DVT = deep vein thrombosis, GCS = Glasgow Coma Scale, hsCRP = high-sensitivity C-reactive protein, LDL-C = low-density lipoprotein cholesterol, INR = international normalized ratio, mRS = modified Rankin scale, NIHSS = National Institutes of Health Stroke Scale, SBP = systolic blood pressure, TOAST = Trial of Org 10172 in Acute Stroke Treatment, VTE = venous thromboembolism.

**Table 2:** Characteristics of train and validation sets (N = 362).

Variable	Category	Train set, N = 253 (%)	Validation set, N = 109 (%)	*P* value
VTE	No	231 (91.30%)	99 (90.83%)	.843
	Yes	22 (8.70%)	10 (9.17%)	
Gender	Male	182 (71.94%)	85 (77.98%)	.244
	Female	71 (28.06%)	24 (22.02%)	
Age	/	58.64 ± 14.38	57.19 ± 14.24	.380
Ethnicity	Han	239 (94.47%)	109 (100.00%)	.007
	Others	14 (5.53%)	0 (0.00%)	
Height	/	165.37 ± 8.61	167.10 ± 6.22	.058
Weight	/	65.06 ± 12.82	67.81 ± 11.35	.054
Wake-up stroke	No	225 (88.93%)	94 (86.24%)	.482
	Yes	28 (11.07%)	15 (13.76%)	
Limb weakness	No	199 (78.66%)	84 (77.06%)	.782
	Yes	54 (21.34%)	25 (22.94%)	
Dysarthria	No	146 (57.71%)	70 (64.22%)	.293
	Yes	107 (42.29%)	39 (35.78%)	
Lmpaired consciousness	No	232 (91.70%)	101 (92.66%)	.836
	Yes	21 (8.30%)	8 (7.34%)	
Headache	No	248 (98.02%)	109 (100.00%)	.328
	Yes	5 (1.98%)	0 (0.00%)	
Dizzy	No	227 (89.72%)	97 (88.99%)	.853
	Yes	26 (10.28%)	12 (11.01%)	
Convulsion	No	251 (99.21%)	108 (99.08%)	.978
	Yes	2 (0.79%)	1 (0.92%)	
Premorbid mRS	0–1	227 (89.72%)	95 (87.16%)	.752
	2–3	14 (5.53%)	7 (6.42%)	
	4–5	12 (4.74%)	7 (6.42%)	
Abnormal electrocardiogram	No	214 (84.58%)	87 (79.82%)	.285
	Yes	39 (15.42%)	22 (20.18%)	
History of cerebral infarction	No	215 (84.98%)	89 (81.65%)	.438
	Yes	38 (15.02%)	20 (18.35%)	
Coronary heart disease	No	236 (93.28%)	102 (93.58%)	.999
	Yes	17 (6.72%)	7 (6.42%)	
Atrial fibrillation	No	230 (90.91%)	99 (90.83%)	.999
	Yes	23 (9.09%)	10 (9.17%)	
DM	No	203 (80.24%)	93 (85.32%)	.300
	Yes	50 (19.76%)	16 (14.68%)	
Hypertension	Yes	139 (54.94%)	62 (56.88%)	.818
	No	114 (45.06%)	47 (43.12%)	
Smoking	No	174 (68.77%)	74 (67.89%)	.902
	Yes	79 (31.23%)	35 (32.11%)	
Alcohol use	No	176 (69.57%)	72 (66.06%)	.538
	Yes	77 (30.43%)	37 (33.94%)	
mRS after admission	0–1	165 (65.22%)	74 (67.89%)	.141
	2–3	76 (30.04%)	25 (22.94%)	
	4–5	12 (4.74%)	10 (9.17%)	.999
Aspirin	No	185 (73.12%)	80 (73.39%)	
	Yes	68 (26.88%)	29 (26.61%)	
Clopidogrel	No	197 (77.87%)	87 (79.82%)	.781
	Yes	56 (22.13%)	22 (20.18%)	
Warfarin	No	243 (96.05%)	104 (95.41%)	.778
	Yes	10 (3.95%)	5 (4.59%)	
Rivaroxaban	Yes	214 (84.58%)	93 (85.32%)	.999
	No	39 (15.42%)	16 (14.68%)	
Sulfonylureas	No	227 (89.72%)	100 (91.74%)	.699
	Yes	26 (10.28%)	9 (8.26%)	
Calcium antagonist	Yes	166 (65.61%)	72 (66.06%)	.999
	No	87 (34.39%)	37 (33.94%)	
Anti-infective	No	215 (84.98%)	87 (79.82%)	.223
	Yes	38 (15.02%)	22 (20.18%)	
Traditional medicine	No	242 (95.65%)	103 (94.50%)	.599
	Yes	11 (4.35%)	6 (5.50%)	
Complications	No	175 (69.17%)	84 (77.06%)	.162
	Yes	78 (30.83%)	25 (22.94%)	
Lipid medicine	No	238 (94.07%)	101 (92.66%)	.641
	Yes	15 (5.93%)	8 (7.34%)	
Antiplatelet therapy	Yes	220 (86.96%)	92 (84.40%)	.511
	No	33 (13.04%)	17 (15.60%)	
Anticoagulant therapy	No	196 (77.47%)	81 (74.31%)	.589
	Yes	57 (22.53%)	28 (25.69%)	
Lipid-lowering therapy	Yes	238 (94.07%)	103 (94.50%)	.999
	No	15 (5.93%)	6 (5.50%)	
Antihypertensive therapy	No	136 (53.75%)	60 (55.05%)	.909
	Yes	117 (46.25%)	49 (44.95%)	
Antidiabetic therapy	No	189 (74.70%)	89 (81.65%)	.175
	Yes	64 (25.30%)	20 (18.35%)	
Intracranial artery stenosis ≥70%	No	172 (67.98%)	79 (72.48%)	.456
	Yes	81 (32.02%)	30 (27.52%)	
Stroke-related pneumonia	No	211 (83.40%)	93 (85.32%)	.755
	Yes	42 (16.60%)	16 (14.68%)	
Post-stroke epilepsy	No	234 (92.49%)	103 (94.50%)	.652
	Yes	19 (7.51%)	6 (5.50%)	
TOAST	Large vessel occlusion	41 (16.21%)	21 (19.27%)	.946
	Small vessel occlusive stroke	106 (41.90%)	44 (40.37%)	
	Cardioembolic stroke	48 (18.97%)	18 (16.51%)	
	Other causes of stroke	51 (20.16%)	23 (21.10%)	
	Unexplained stroke	7 (2.77%)	3 (2.75%)	
NIHSS after onset	0–4	61 (24.11%)	29 (26.61%)	.904
	5–14	145 (57.31%)	62 (56.88%)	
	15–20	29 (11.46%)	10 (9.17%)	
	21–42	18 (7.11%)	8 (7.34%)	
NIHSS during hospitalization	0–4	64 (25.30%)	31 (28.44%)	.863
	5–14	131 (51.78%)	56 (51.38%)	
	15–20	40 (15.81%)	14 (12.84%)	
	21–42	18 (7.11%)	8 (7.34%)	
GCS	13–15	190 (75.10%)	84 (77.06%)	.919
	9–12	36 (14.23%)	14 (12.84%)	
	3–8	27 (10.67%)	11 (10.09%)	
SBP	/	151.57 ± 25.42	153.50 ± 28.13	.520
DBP	/	91.01 ± 15.06	90.85 ± 18.76	.932
White blood cell count	/	9.14 ± 4.00	8.30 ± 2.79	.047
Neutrophil count	/	6.51 ± 4.49	5.70 ± 2.92	.084
Lymphocyte count	/	2.07 ± 1.11	1.91 ± 1.01	.218
hsCRP	/	9.81 ± 18.23	8.88 ± 14.79	.638
INR	/	1.05 ± 0.22	1.15 ± 1.14	.217
Fibrinogen	/	3.08 ± 0.78	3.13 ± 0.87	.619
D-dimer	/	175.24 ± 1659.91	93.65 ± 622.18	.619
Fasting blood glucose	/	6.46 ± 2.41	6.33 ± 2.49	.620
Glycosylated hemoglobin	/	6.48 ± 1.80	6.16 ± 1.18	.091
Alanine aminotransferase	/	24.58 ± 23.50	25.99 ± 14.29	.562
LDL-C	/	2.91 ± 0.97	2.97 ± 0.86	.562
Serum uric acid	/	389.00 ± 115.35	409.83 ± 132.20	.133
Serum creatinine	/	83.60 ± 56.27	82.60 ± 33.10	.862
Urea nitrogen	/	7.61 ± 21.24	6.28 ± 5.02	.518
Homocysteine	/	14.79 ± 7.19	17.76 ± 11.54	.003

Categorical variables are presented as n (%) and compared using chi-square or Fisher exact tests. Continuous variables are presented as mean ± standard deviation and compared using *t* tests or Mann–Whitney *U* tests. *P* < .05 was considered statistically significant.

DM = diabetes mellitus, DBP = diastolic blood pressure, GCS = Glasgow Coma Scale, hsCRP = high-sensitivity C-reactive protein, LDL-C = low-density lipoprotein cholesterol, INR = international normalized ratio, mRS = modified Rankin scale, NIHSS = National Institutes of Health Stroke Scale, SBP = systolic blood pressure, TOAST = Trial of Org 10172 in Acute Stroke Treatment, VTE = venous thromboembolism.

## 4.2. Comparison between DVT and non-DVT groups

Among the baseline characteristics, several variables showed statistically significant differences between the DVT and non-DVT groups. Patients who developed DVT were more likely to have undergone endovascular thrombectomy (62.50% vs 37.58%, *P* = .008) and less likely to be of Han ethnicity (87.50% vs 96.97%, *P* = .027). They also had higher incidences of limb weakness (43.75% vs 18.48%, *P* < .001) and dysarthria (84.38% vs 36.06%, *P* < .001). In addition, DVT patients had significantly more frequent headaches (9.38% vs 0.61%, *P* = .006), higher premorbid mRS scores (*P* < .001), and more abnormal electrocardiogram results (40.62% vs 14.55%, *P* = .001; Table 1).

## 4.3. Feature importance analysis

**Figure 1.**
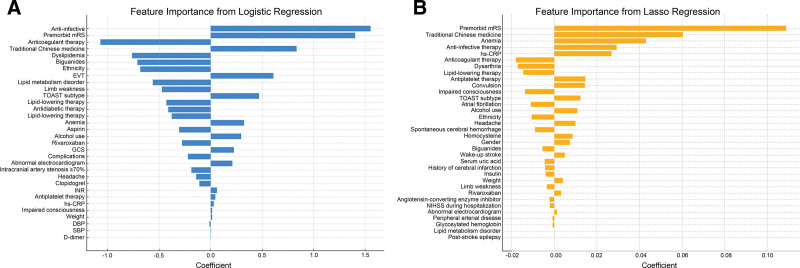
Feature importance from logistic and LASSO regression. (A) Logistic regression feature importance; (B) LASSO regression feature importance. DBP = diastolic blood pressure, EVT = endovascular thrombectomy, GCS = Glasgow Coma Scale, hsCRP = high-sensitivity C-reactive protein, INR = international normalized ratio, mRS = modified Rankin scale, SBP = systolic blood pressure.

The feature importance for predicting the risk of DVT in AIS patients undergoing revascularization therapy was derived from both LASSO and logistic regression methods. LASSO regression identified premorbid mRS scores, D-dimer, anemia, anti-infective treatments, high-sensitivity C-reactive protein (hsCRP) levels, and in-hospital anticoagulant use as the most significant predictors (Fig. 1A). In parallel, logistic regression highlighted anti-infective therapy, premorbid mRS scores, in-hospital anticoagulant use, traditional medicine, and dysarthria as key factors, alongside contributions from biguanides, ethnicity, and EVT (Fig. 1B). These analyses underscore the multifactorial nature of DVT risk, revealing several overlapping and distinct predictors. Such insights are invaluable for clinical decision-making and inform targeted interventions to mitigate DVT risk in this patient population.

## 4.4. SMOTE and t-distributed stochastic neighbor embedding visualization

**Figure 2.**
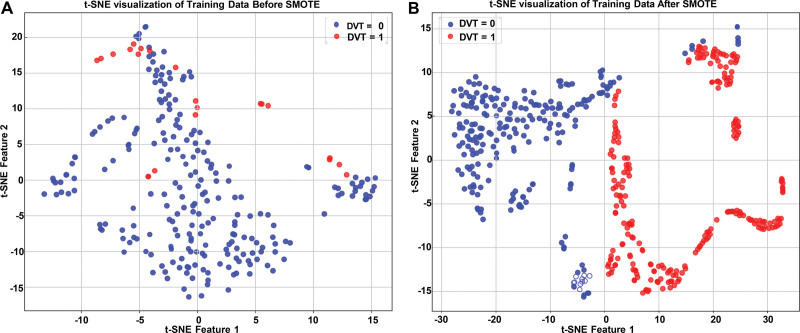
The t-SNE visualization before and after SMOTE. (A) t-SNE plot of training data before SMOTE, (B) t-SNE plot of training data after SMOTE. DVT = deep vein thrombosis, SMOTE = synthetic minority over-sampling technique, t-SNE = t-distributed stochastic neighbor embedding.

To address class imbalance, we applied the SMOTE to the training data. The t-distributed stochastic neighbor embedding visualization of the data before and after SMOTE application demonstrates its impact. Initially, DVT cases were sparse and scattered (left panel, Fig. 2). Post-SMOTE, DVT cases were significantly increased and more evenly distributed, ensuring better representation in the training set (right panel, Fig. 2). This balanced distribution enhances the model’s ability to effectively learn from both classes.

## 4.5. Model performance

**Figure 3.**
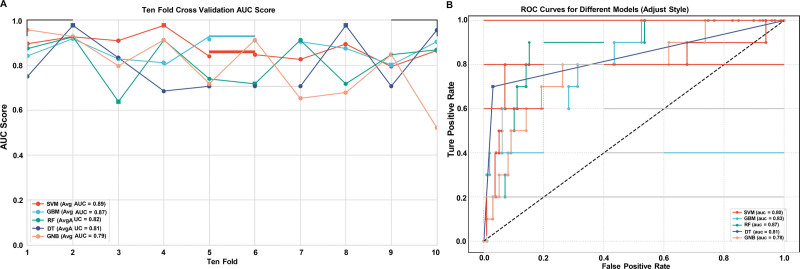
Model performance and ROC curves. (A) Ten-fold cross-validation AUC scores, (B) ROC curves for different models on the test set. AUC = area under the curve, ROC = receiver operating characteristic.

RF and GBM models demonstrated superior performance during 10-fold cross-validation, with average AUC scores of 0.82 and 0.87, respectively (Fig. 3A). When evaluated on the test set, RF maintained the highest performance (AUC = 0.87), followed by GBM (AUC = 0.83), DT (AUC = 0.81), SVM (AUC = 0.80), and Gaussian naive Bayes (AUC = 0.78; Fig. 3B). The supplementary analysis revealed that RF achieved the highest specificity (0.93) and accuracy (0.87), with sensitivity of 0.86. GBM showed comparable performance with specificity of 0.91 and accuracy of 0.84. In contrast, SVM exhibited relatively high sensitivity (0.85) but lower specificity (0.66) and accuracy (0.68). The ROC curves (Fig. 3C) confirmed that RF provided optimal discrimination between DVT-positive and DVT-negative cases across all evaluated models. Detailed performance results are provided in Table S1.

## 4.6. Model interpretation with SHAP

**Figure 4.**
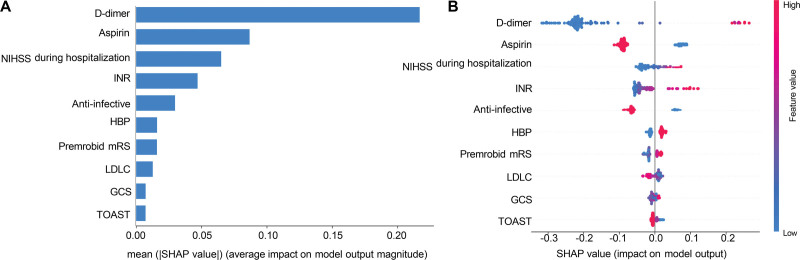
SHAP analysis of the RF model. (A) SHAP summary plot; (B) SHAP bar chart of feature importance. GCS = Glasgow Coma Scale, HBP = high blood pressure, INR = international normalized ratio, LDL-C = low-density lipoprotein cholesterol, mRS = modified Rankin scale, RF = random forest, SHAP = SHapley Additive exPlanations, SBP = systolic blood pressure, TOAST = Trial of Org 10172 in Acute Stroke Treatment.

The SHAP-based interpretation of the best-performing model provides insights into the impact of individual features on DVT risk predictions. The SHAP summary plot (Fig. 4A) shows that D-dimer, aspirin, and NIHSS during hospitalization have the highest SHAP values, indicating their significant influence on the model’s predictions. Other critical features include international normalized ratio (INR), anti-infective treatments, high blood pressure, premorbid mRS, low-density lipoprotein cholesterol, GCS score, and Trial of Org 10172 in Acute Stroke Treatment classification. The dots represent SHAP values for individual instances, with colors indicating the feature value’s magnitude, providing a comprehensive view of each feature’s impact across the dataset. Complementing this, Figure 4B displays a bar chart of the mean absolute SHAP values, ranking the features by their average impact on model output. Consistent with the summary plot, D-dimer, aspirin, and NIHSS during hospitalization are identified as the top 3 features, followed by INR and anti-infective treatments. This bar chart succinctly summarizes the relative importance of each feature, reinforcing the critical role these variables play in the model’s decision-making process.

## 5. Discussion

AIS poses significant public health challenges, with complications such as DVT severely impacting patient outcomes. Traditional prediction methods for DVT in AIS patients undergoing revascularization therapy often lack accuracy. In this study, we addressed this issue by developing and validating ML models, with the RF model achieving the highest performance (AUC of 0.87). Key predictors included D-dimer levels, antiplatelet medications (aspirin and NIHSS score), INR, and anti-infective treatments. The use of SHAP enhanced model interpretability, making the predictions more transparent and clinically actionable. By providing early and accurate risk assessments, these ML models enable timely prophylactic interventions, potentially reducing morbidity and mortality in AIS patients post-revascularization. The integration of such advanced predictive models into clinical practice could significantly improve stroke care and patient outcomes.

Our findings build upon and extend previous research on DVT prediction in stroke patients. For instance, Pan et al developed a nomogram with an AUC of 0.785 for predicting distal DVT within 14 days of stroke onset.^[14]^ Similarly, other studies reported AUC values between 0.756 and 0.820 using traditional statistical methods.^[15,16]^ In contrast, our study employs advanced ML techniques, specifically the RF algorithm, which demonstrated superior predictive accuracy with an AUC of 0.87. This higher accuracy is achieved through the ML model’s ability to analyze high-dimensional data and identify complex interactions among variables, which traditional statistical methods might miss. In the broader context of AIS prognosis, several studies have highlighted the superior performance of RF algorithms. For example, Heo et al reported that RF outperformed other ML models in predicting long-term outcomes in AIS patients, demonstrating higher accuracy and robustness.^[17]^ Our findings are consistent with this trend, with the RF model achieving the highest AUC, underscoring its efficacy in capturing the multifactorial nature of DVT risk. Furthermore, studies by Bonkhoff et al have shown that integrating ML methods, including RF, can enhance predictive accuracy, although these studies did not focus specifically on AIS patients post-revascularization.^[18]^ This study advances the field by demonstrating the superior predictive power of ML models compared with traditional statistical approaches. Traditional models often rely on a limited set of predictors and linear relationships, which can overlook complex patterns in the data. In contrast, ML models such as RF and gradient boosting classifiers can capture nonlinear interactions and dependencies among predictors, providing a more nuanced and accurate risk assessment.^[19]^ Moreover, the use of interpretative tools like SHAP enhances the transparency of ML models, making their predictions more understandable and actionable for clinicians. This addresses a common criticism of ML models as “black boxes” and promotes their integration into clinical practice.

To delve deeper into the predictors identified by our model, the SHAP analysis showed that D-dimer was the most significant predictor, consistent with previous studies highlighting its role in DVT risk.^[20]^ Elevated D-dimer levels are associated with an increased risk of recurrent stroke and DVT after AIS, emphasizing the importance of early detection and management. In addition, the NIHSS score during hospitalization and the premorbid mRS score emerged as crucial predictors. High NIHSS scores correlate with worse outcomes and a higher risk of stroke recurrence, necessitating their inclusion in risk assessment protocols.^[21]^ This metric helps clinicians identify patients requiring more intensive monitoring and tailored interventions during and after hospitalization. Similarly, the premorbid mRS score, which measures the degree of disability before the stroke, significantly contributes to the model. Patients with higher premorbid disability are at greater risk of recurrence, underscoring the importance of comprehensive pre-stroke health assessments to determine post-stroke care plans.^[22]^ Low-density lipoprotein cholesterol levels were also significant, underscoring the need for effective lipid-lowering therapies to mitigate the risk of atherosclerosis and subsequent strokes.^[23]^ By integrating these findings, our study not only enhances the predictive accuracy for DVT in AIS patients but also provides actionable insights for clinical practice. The robust performance of our RF model, supported by the interpretability offered by SHAP, highlights the potential for ML models to transform DVT risk assessment and management in stroke care.

Building on these insights, the prediction models developed in this study hold substantial potential for clinical practice. By accurately stratifying patients according to DVT risk, these models can guide timely preventive interventions, reducing morbidity and mortality associated with DVT and related complications such as pulmonary embolism.^[24]^ Given their basis in routine clinical and laboratory data, these models can be seamlessly integrated into electronic health records and clinical decision support systems.^[25]^ This integration would enable real-time risk assessment, providing clinicians with immediate feedback and recommendations. Consequently, such systems can enhance proactive management of high-risk patients, streamline clinical workflows, reduce the burden on healthcare providers, and ultimately improve patient outcomes.

This study showcases several key strengths. First, it employs advanced ML techniques, including RF, GBM, and SVM, which enhance the predictive accuracy by capturing complex interactions among variables. Second, the interpretability of the models is significantly enhanced through the use of SHAP. These tools provide transparent insights into how individual predictors contribute to the risk predictions, thereby fostering trust and facilitating informed clinical decision-making. Third, the study focuses specifically on AIS patients post-revascularization, a high-risk subgroup with unique physiological and procedural risk factors, ensuring that the model is highly relevant to this critical patient population. In addition, the final features included in the model are clinically accessible variables, making the model practical and easy to implement in real-world settings. Lastly, the study follows the PROBAST guidelines to avoid bias, ensuring the methodological rigor and reliability of the findings.^[26]^ These strengths collectively highlight the study’s robust approach to improving DVT risk prediction in AIS patients and its potential for integration into clinical practice.

## 6. Conclusion

This study developed and validated advanced ML models, particularly an RF algorithm, to predict DVT risk in AIS patients undergoing revascularization therapy. The RF model outperformed traditional methods, with key predictors including D-dimer, aspirin, NIHSS during hospitalization, INR, and anti-infective. The SHAP enhanced model interpretability, making predictions more actionable for clinicians. Integrating these models into electronic health records and clinical decision support systems can enable real-time risk assessments and timely interventions, reducing morbidity and mortality. This approach shows significant potential for improving DVT risk prediction and patient outcomes in AIS care, warranting further validation in larger, multicenter cohorts.

## Author contributions

**Conceptualization:** Yanfeng Li, Rong Li.

**Methodology:** Youli Jiang, Yun Han, Rongjia Pan.

**Resources:** Youli Jiang.

**Investigation:** Min Gui, Rongjia Pan.

**Validation:** Yun Han.

**Data curation:** Guisu Li.

**Formal analysis:** Guisu Li, Qingshi Zhao.

**Project administration:** Qingshi Zhao.

**Supervision:** Qingshi Zhao.

**Writing – review & editing:** Youli Jiang, Qingshi Zhao.


