# Supplementary material for: Explainable AI models for predicting venous thromboembolism following revascularization therapy in ischemic stroke patients: A retrospective cohort study
Source: Medicine (Baltimore). 2026 May 15;105(20):e48657. doi: 10.1097/MD.0000000000048657 (PMC13183046; doi:10.1097/MD.0000000000048657)
Supplement: Supplementary file 1 [file medi-105-e48657-s001.docx]

**Supplementary Table S1. Comprehensive Evaluation Metrics of Different Machine Learning Algorithms in the DVT Prediction Model.**

| **Model** | **AUC of test set** | **Sensitivity** | **Specificity** | **Accuracy** |
| --- | --- | --- | --- | --- |
| RF | 0.87 | 0.86 | 0.93 | 0.87 |
| GBM | 0.83 | 0.81 | 0.91 | 0.84 |
| GNB | 0.78 | 0.81 | 0.85 | 0.83 |
| DT | 0.81 | 0.80 | 0.82 | 0.82 |
| SVM | 0.80 | 0.85 | 0.66 | 0.68 |
